# MicroRNAs and senescence

**DOI:** 10.18632/aging.100282

**Published:** 2011-02-07

**Authors:** Ivan Martinez, Laura L. Almstead, Daniel DiMaio

**Affiliations:** Department of Genetics, Yale University School of Medicine, New Haven, CT 06510, USA

The discovery and analysis of microRNAs (miRNAs) has revealed a new mechanism of gene regulation. miRNAs can base-pair with specific mRNA targets and regulate their expression at the post-transcriptional level [1]. Changes in miRNA abundance are implicated in controlling many biological processes including cellular senescence [2], an irreversible form of growth arrest involved in cellular aging. The p53 and retinoblastoma (Rb) tumor suppressor pathways are essential for the establishment and maintenance of growth arrest during senescence [3], reflecting the need of cells to bypass senescence during carcinogenesis.

Multiple miRNAs have been implicated in regulating the p53 pathway during cellular senescence. For example, miR-217 and the miR-34 family target the *SIRT1* deacetylase gene. Reduction of *SIRT1* expression by these miRNAs permits the maintenance of p53 acetylation, resulting in p53 stabilization and the induction of senescence [4,5]. In mouse embryonic fibroblasts, miR-20a represses the expression of the transcriptional regulator LRF (Leukemia/Lymphoma Related Factor), a repressor of the p19ARF gene. The absence of LRF increases the expression of p19ARF, which in turn inhibits the expression of the ubiquitin ligase MDM2, causing up-regulation of p53 and induction of senescence [6].

Our group determined which miRNAs were differentially expressed during Rb-induced senescence, and we explored the roles of some of these miRNAs in this process [7]. We analyzed the global expression of miRNAs in an established model of cellular senescence in which repression of the human papillomavirus E7 oncogene causes the rapid induction of Rb-dependent senescence in HeLa cervical carcinoma cells [8,9]. We found that 25 miRNAs were up-regulated and 24 were down-regulated in an Rb-dependent manner during induced senescence.

Notably, several members of the miR-29 and miR-30 families were up-regulated during senescence, and this up-regulation required activation of the Rb pathway. Further analysis showed that miR-29 and miR-30 target the 3' untranslated region (3' UTR) of *B-Myb* mRNA [7]. The *B-Myb* oncogene (also known as *MYBL2*) encodes a transcription factor that regulates genes involved in control of the cell cycle, and modulation of *B-Myb* expression is known to influence senescence [10,11,12]. The interaction of miR-29 and miR-30 with the *B-Myb* 3' UTR inhibits gene expression during both induced and replicative senescence. *B-Myb* expression can also be repressed by direct binding of Rb-E2F factors to the *B-Myb* promoter. Interestingly, over-expression of miR-29 and miR-30 in proliferating cells suppresses *B-Myb* expression and cellular DNA synthesis, and inhibiting the expression of these miRNAs allowed a population of cells to escape growth arrest caused by HPV E7 repression [7]. Based on these results, we concluded that miR-29 and miR-30 play an important role during Rb-induced senescence.

miR-29 and miR-30 were previously implicated as potential tumor suppressor factors. For example, miR-29 can inhibit DNA methylation in lung cancer cells by targeting DNA methyl-transferase 3A and 3B expression and suppress the growth of these cells [13]. miR-29 can also repress expression of the T-cell leukemia/lymphoma 1 (tcl1) oncogene and may act as a tumor suppressor in chronic lymphocytic leukemia [14]. In addition, miR-29 was recently shown to repress expression of cdc42 and the p85 subunit of PI-3' kinase, which in turns results in the stabilization of p53 and apoptosis [15]. miR-29 expression can also induce apoptosis in cholangiocarcinoma cells by down-regulating expression of the anti-apoptotic protein Mcl-1 [16]. Finally, a recent study showed that exogenous expression of miR-29a in lung and pancreatic cancer cell lines reduced proliferation and invasiveness in vitro. This group also used proteomics analysis to identify around 100 differentially-expressed proteins as potential targets of miR-29a [17]. miR-30 expression is down-regulated in human breast, head-and-neck, and lung cancer in comparison to normal tissue, and transfection of miR-30 decreased cell proliferation by targeting Ubc9, an enzyme involved in sumoylation [18]. Our finding that miR-29 and miR-30 inhibit *B-Myb* expression suggests that *B-Myb* repression may also contribute to the tumor suppressor activities of these miRNAs.

We also investigated miRNA expression during replicative senescence in primary cells, a process that involves p53 as well as Rb. Twenty-two miRNAs were affected during both replicative senescence in primary human foreskin fibroblasts and Rb-induced senescence in HeLa cells. Remarkably, 20 of these 22 miRNAs (including some miR-29 and miR-30 family members) were regulated in the same direction in both types of senescence, whereas the abundance of only two miRNAs changed opposite directions, suggesting that the core Rb/miRNA circuit plays a similar role in induced and replicative senescence. In addition, a number of miRNAs were found to be induced or repressed during replicative senescence but not during Rb-induced senescence, suggesting that they may be targets of p53-mediated regulation.

These findings raise a number of interesting questions. Are additional miRNAs involved in controlling irreversible growth arrest in replicative and induced senescence? Do the same miRNAs regulate induced and replicative senescence? Do different miRNAs regulate senescence in different tissues and cell types? Most importantly, which genes are targeted by these miRNAs and how do the products of these genes contribute to senescence? Further studies are likely to answer these questions and clarify the role of miRNAs in irreversible growth arrest and carcinogenesis.
